# Microgeographic morphological variation across larval wood frog populations associated with environment despite gene flow

**DOI:** 10.1002/ece3.3829

**Published:** 2018-02-01

**Authors:** Amanda J. Zellmer

**Affiliations:** ^1^ Department of Biology Occidental College Los Angeles CA USA

**Keywords:** common garden, gene flow, local adaptation, plasticity, *Rana sylvatica*

## Abstract

Gene flow has historically been thought to constrain local adaptation; yet, recent research suggests that populations can diverge despite exchanging genes. Here I use a common garden experiment to assess the combined effects of gene flow and natural selection on morphological variation of 16 wood frog (*Rana sylvatica*) populations, a species known to experience divergent selection pressures in open‐ and closed‐canopy ponds across relatively small geographic scales. Wood frog tadpoles from different ponds showed significant morphological variation associated with canopy type with a trade‐off between tail length and body depth consistent with previous research. In contrast, neutral genetic differentiation of nine microsatellite loci as measured by Jost's *D* was not associated with canopy type, indicating no pattern of isolation by environment. Genetic structure analyses indicated some substructure across the 16 ponds (*K *=* *4); however, three out of four assigned clusters included both open‐ and closed‐canopy ponds. Together, these results suggest that morphological divergence among these wood frog populations is occurring despite gene flow and that selection within these environments is strong. Furthermore, morphological variation among ponds differed across two sampling periods during larval development, demonstrating the importance of evaluating phenotypic divergence over multiple time periods and at a time relevant to the processes being studied.

## 1 INTRODUCTION

Gene flow has historically been thought to counteract the effects of selection, preventing local adaptation and leading to the homogenization of populations (Bridle & Vines, 2007; Kawecki & Ebert, 2004; Lenormand, 2002; Slatkin, 1985). This pattern has been demonstrated across a wide range of taxa (see Rasanen & Hendry, 2008). Yet, recent research suggests that populations may diverge in the absence of strong physical barriers to gene flow (Emelianov, Marec, & Mallet, 2004; Jordan, Snell, Snell, & Jordan, 2005; Kotlík et al., 2008; Larsen et al., 2007; McCormack & Smith, 2008; Niemiller, Fitzpatrick, & Miller, 2008; Nosil, Crespi, Sandoval, & Kirkpatrick, 2006; Rice & Hostert, 1993; Schneider, Smith, Larison, & Moritz, 1999; Smith, Wayne, Girman, & Bruford, 1997), even at microgeographic scales (Richardson, Urban, Bolnick, & Skelly, 2014). Gene flow may even facilitate the divergence of populations by providing the genetic variation necessary for selection to act upon (Rieseberg & Burke, 2001). This paradox arises in part because each gene differs in the extent to which it is affected by the interplay between gene flow and selection. Genomes are heterogeneous (Wu, 2001), with the strength of selection and rate of introgression varying across different genes. As a result, genes coding for phenotypic traits under intense selection pressures may diverge between populations, whereas nonselected genes become homogenized (Nosil, Egan, & Funk, 2008). Understanding how this interplay between gene flow and selection affects adaptation is crucial, as the extent to which populations can become locally adapted and phenotypically divergent is important for understanding the processes of divergence and speciation. Further, knowing the relative roles of gene flow and selection is necessary for predicting the potential for populations to adapt and cope with human‐induced environmental changes following reduced gene flow due to habitat loss and fragmentation.

In this study, I sought to assess the contribution of gene flow and selection to local adaptation of the wood frog, *Rana sylvatica,* a North American amphibian that inhabits a relatively broad environmental gradient, with larvae occupying both open‐ and closed‐canopy ponds (Werner & Glennemeier, 1999). Open‐canopy ponds on average have greater resource availability, have higher dissolved oxygen levels, have longer hydroperiods, are warmer than closed‐canopy ponds (Werner & Glennemeier, 1999), and also harbor more predators (Relyea, 2002b), whereas closed‐canopy ponds—due to resource scarcity—have higher levels of intraspecific competition (Werner, Skelly, Relyea, & Yurewicz, 2007). As a result, selection pressures in open‐ versus closed‐canopy ponds are strongly divergent. Selection by predators favors individuals that invest in antipredator defenses, including reduced activity and increased tail development, at the expense of decreased growth rates (Relyea, 2000, 2001a,b; Relyea & Werner, 2000; Van Buskirk, 2002; Van Buskirk, McCollum, & Werner, 1997; Van Buskirk & Relyea, 1998). In contrast, intense competition (or low resource levels) favors individuals that maximize growth rates as opposed to antipredator defenses (Relyea, 2002a). In fact, previous research has demonstrated distinct morphological differences among ponds in common garden and reciprocal transplant experiments, suggesting that these selection pressures promote the adaptation of wood frog populations to local environmental conditions (Relyea, 2002b; Skelly, 2004).

While these population‐level differences were initially attributed to isolation of ponds due to limited dispersal (Relyea, 2002b), more recent research suggests that amphibians have greater dispersal capabilities than previously thought (Smith & Green, 2005). Since the alternative habitats are interspersed across the landscape, with open‐ and closed‐canopy ponds well within the dispersal capabilities of wood frogs (Berven & Grudzien, 1990), there is potential for gene flow to occur among opposing selective regimes. Thus, the phenotypic differences among ponds may be due to either environmental barriers preventing gene flow among populations (Wang & Bradburd, 2014) or strong selection on maladapted individuals (Relyea, 2002b). Although local adaptation and phenotypic variation of wood frogs in response to environmental variation have long been studied through both experimental research and natural history observations (Relyea, 2000, 2001a,b; Relyea & Werner, 2000; Van Buskirk, 2002; Van Buskirk & Relyea, 1998; Van Buskirk et al., 1997), the genetics of this variation and contribution of gene flow to these phenotypic differences has yet to be assessed.

To determine the relative roles of gene flow and local environmental conditions on phenotypic variation, I conducted a common garden experiment paired with an evaluation of population genetic structure of wood frog larvae from 16 ponds with divergent environments. Since environmental differences are thought to promote local adaptation, I expect morphological differences between open‐ and closed‐canopy ponds, consistent with previous research (Relyea, 2002b). However, this variation may also be impacted by the amount of gene flow occurring among ponds. I thus investigated the extent to which gene flow occurs among ponds and among canopy types and tested for an association between gene flow and the extent of morphological differences among ponds. If morphological variation is the result of strong selective differences among ponds despite gene flow, then there should be a significant difference in morphology among open‐ and closed‐canopy ponds with evidence of gene flow. If on the other hand selection influences gene flow, then I expect a significant pattern of isolation by environment, with neutral genetic divergence increasing with environmental differences. I evaluated these hypotheses at two separate time periods during larval development, while larvae are more or less susceptible to predation by gape‐limited predators, to determine whether the effects were lasting beyond the selection period. The traits evaluated include mass and five morphological measurements: tail length and body depth, which are heritable and under selection by predators (Relyea, 2005; Van Buskirk & Relyea, 1998), tail depth and body length, which have heritable plasticity (Relyea, 2005), and muscle depth, which is heritable (Relyea, 2005) but has little evidence of being under selection by *Anax* predators (Van Buskirk & Relyea, 1998).

## 2 METHODS

To test hypotheses about the role of gene flow and environment on morphological divergence, I paired a common garden mesocosm experiment with an analysis of environmental and genetic variation of the original populations. I conducted a common garden experiment to quantify the morphological variation among ponds independent of environmental influences. To quantify environmental differences, I assessed pond canopy cover of each of the populations. To assess genetic divergence, I used microsatellite data to quantify pairwise genetic differences as well as population genetic structure. Finally, I compared morphological differences among ponds in relation to canopy and genetic cluster.

### 2.1 Study species

The wood frog (*Rana sylvatica*) is a pond‐breeding amphibian that inhabits much of eastern North America, with a range extending up to Alaska. The wood frog is an explosive breeder that lays eggs during a 1–2 week period in the spring. Females usually lay their eggs in a single egg mass consisting of on average 711 eggs (Benard, 2015), and most females from a single breeding chorus lay their egg masses next to one another. Breeding populations are therefore usually discrete units. Eggs take approximate 1–2 weeks to hatch. Tadpoles grow in the ponds for about 6 weeks and then after metamorphosis, enter forested habitat.

### 2.2 Common garden experiment

**Figure 1:**
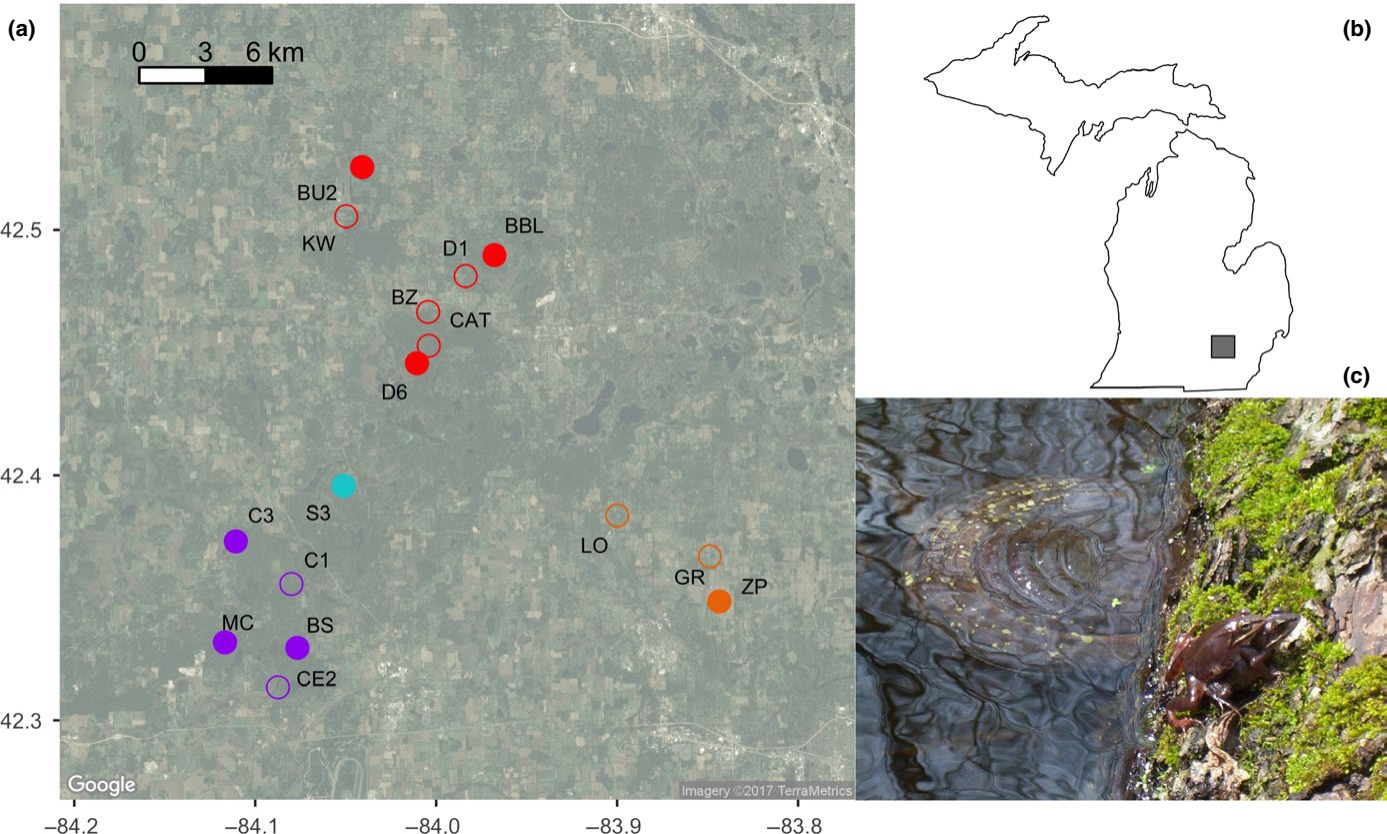
Map of study area of *Rana sylvatica* populations. (a) Map of study area in southeastern Michigan with satellite image showing habitat in background. Open‐canopy (open circles) and closed‐canopy (solid circles) ponds. Colors indicate genetic clusters as assigned by structure (*K *=* *4). (b) Inset shows location of study area in southeastern Michigan, USA. (c) Amplectant wood frogs in open‐canopy Cassidy 1 (C1) pond

To quantify morphological differences among wood frog populations, I conducted a common garden experiment raising individuals from 16 ponds that were classified as either open‐ or closed‐canopy in mesocosms with similar conditions. Ponds were selected to ensure that dispersal and gene flow were possible among populations. Each of the populations used in this study were within the average wood frog dispersal distance (approximately 1.2 km: Berven & Grudzien, 1990) from at least one other pond and were either within or (in one case) just beyond the maximum‐recorded dispersal distance (2.53 km: Berven & Grudzien, 1990) from at least one other pond of the opposite canopy type (Figure 1). Moreover, some ponds were within less than 165 m from a pond of the opposite habitat type.

During the 2008 breeding season, 16 ponds were visited routinely to determine breeding chorus locations. Approximately 100 eggs were collected from each of 10 egg masses from each pond to ensure a broad sampling of the population. For one pond (Cassidy 1), egg masses could not initially be located, and instead approximately 15 amplectant pairs were caught, kept separate, and returned to the laboratory to breed. Eggs from 10 masses (laid within 24 hours of collection) were kept for this study, and the adults were returned to their pond of origin. Egg masses were later located to confirm that breeding did occur in this pond. All eggs were kept until hatching in outdoor wading pools covered by shade cloth.

Individuals were raised in common garden mesocosms (1000 L polyethylene cattle watering tanks). Each population was raised in separate tanks replicated four times across four spatial blocks, for a total of 64 tanks (16 populations × 4 blocks). The mesocosms were set up 16–21 April, 2008. Each tank was filled with aged well water, inoculated with zooplankton and approximately 6 L of filtered pond water to initiate phytoplankton growth, supplemented with approximately 300 g of leaves to serve as a substrate for phytoplankton, and covered with shade cloth to prevent colonization by other frogs or aquatic predators. Each tank was supplemented with approximately 30 g of rabbit chow on 24 April.

On 22 April, 420 hatchlings were haphazardly selected from each of the 16 populations and kept overnight in containers in the laboratory. Twenty of the hatchlings from each pond were set aside in separate containers for approximately 24 h to assess effects of handling on mortality at stocking. There was 100% survivorship across all ponds during this period. These twenty hatchlings per pond were then preserved in 10% formalin for morphological measurements. The experiment was initiated on 23 April, with 100 hatchlings added to each tank.

### 2.3 Morphological measurements

On days 18 and 37, ten tadpoles were removed from each tank and preserved in 10% formalin, for a total of 40 individuals per population. The sampling dates were chosen for comparison to previous studies (Relyea, 2002b) and to evaluate changes over time. At day 18, tadpoles were still small enough to be susceptible to gape‐limited predation, whereas at day 37, tadpoles should be large enough to be outside the limits of many predators. It is important to note that they may still be susceptible to gape‐unlimited predators. All tadpoles collected were photographed and weighed. Morphological measurements were made on the photographs using ImageJ software. Since the effects of selection and gene flow can vary across the genome, phenotypic measurements were made on a number of traits that are heritable (Relyea, 2005) and under selection by aquatic predators (Van Buskirk & Relyea, 1998). Five morphological measurements were made on each individual, including body length, tail length, body depth, tail depth, and muscle depth (see Relyea, 2000). While geometric morphometric methods provide additional information over linear measurements (Rohlf & Marcus, 1993), linear measurements were used because previous studies have identified adaptive differences based on linear measurements in wood frog larvae. Consistency with these previous studies allows for direct tests of hypotheses generated from previous work. All phenotypic measurements were conducted without knowledge of the source population to reduce the possibility for bias in the measurements.

The morphological measurements were ln‐transformed for linearity. All statistical analyses were conducted in R (R Core team 2015). After averaging across individuals within tanks for each time period, I conducted a nested, repeated‐measures ANCOVA to test for significant variation for each trait across ponds and over time taking into account any differences among spatial blocks. I included ln‐transformed mass to control for differences due to body size. *P*‐values were corrected using a sequential Bonferroni correction (Rice, 1989). To facilitate pairwise comparisons, morphological measurements were also averaged across tanks to a single value for each pond at each sampling period. These pond averages were used for all subsequent analyses. To quantify a combined measure of morphological differences among ponds, I created a morphological distance matrix using the R “vegan” package (Oksanen, Blanchet, Kindt, Legendre, & O'Hara, 2016) for each sampling period.

### 2.4 Environmental differences

Each pond was classified as either open‐ or closed‐canopy as a proxy for predator levels. Since invertebrate predator population sizes fluctuate widely from year to year (Van Buskirk & Relyea, 1998), canopy is a more reliable predictor. Data from the long‐term ecological survey of amphibian and invertebrate populations on the Edwin S. George Reserve at the University of Michigan (Werner et al. unpublished data) demonstrate a significant negative correlation between the amount of canopy cover and the density of invertebrate predators (linear regression: *R* ^*2*^ = 0.461, *p *<* *.014) over an 11‐year period. Canopy was calculated as percent of light transmission through the canopy using a fish‐eye lens from a camera placed on the surface of the water in the center of each pond during June 16–18. Open‐canopy ponds had on average 80.4% light transmission, whereas closed‐canopy ponds had on average 29.9% light transmission (Welch's t‐test: *t *=* *8.36, *p *<* *1.17 × 10^−6^).

### 2.5 Genetic divergence

To quantify the extent of gene flow among ponds, I evaluated genetic population structure among the 16 ponds by using a clustering analysis to detect genetic clusters and by calculating pairwise genetic differentiation. Both measures of genetic differentiation were based on data from nine microsatellite loci from approximately 20 individuals per population published in a previous study (Zellmer & Knowles, 2009).

To test for genetic clusters, I used STRUCTURE v 2.3.4 (Pritchard, Stephens, & Donnelly, 2000). I tested whether there was detectable genetic structure across the 16 ponds and if so whether that structure was related to selection regime (open‐ versus closed‐canopy ponds) or to individual ponds. Three analyses were performed using 1) no prior information, 2) individual ponds as sampling location prior, 3) open‐ and closed‐canopy as a sampling location prior. For each analysis, all other default settings were used with a burnin of 5 × 10^6^ and 1 × 10^6^ iterations to assure alpha converged in each run. For each set of analyses, *K* was set from 1–17 and was replicated three times for each *K*. Following the STRUCTURE analyses, I evaluated the data using Structure Harvester (Earl & vonHoldt, 2012) and CLUMPP (Jakobsson & Rosenberg, 2007) via CLUMPAK (Kopelman, Mayzel, Jakobsson, & Rosenberg, 2015). The highest likelihood and the max Δ*K* value (Evanno, Regnaut, & Goudet, 2005) in addition to histograms of population assignment values were each used to identify the number of clusters with the best support.

Genetic differentiation was calculated as *D*_*est*_ (Hedrick, 1999; Jost, 2008) using the R “diveRsity” package (Keenan, Mcginnity, Cross, Crozier, & Prodöhl, 2013), since *D*_*est*_ is less susceptible to gene variation resulting in a better estimator of allelic differentiation among populations as compared to *G*_*ST*_ values (Jost, 2008). Populations were considered diverged if the 95% confidence intervals for Jost's *D* did not overlap 0.

To determine whether gene flow is limited by environmental difference, I conducted an Isolation by Environment (IBE) analysis (Wang, 2013). IBE tests whether gene flow is limited by differences in the environment controlling for geographic distances separating ponds. To test for IBE, I used a multiple matrix regression technique (MMRR; Wang, 2013). MMRR uses permutation tests to evaluate correlations of multiple predictor matrices (geographic distance and environment) with the response variable matrix (genetic distance). Gene flow was measured as pairwise Jost's *D* among ponds. Geographic distances were calculated as Haversine distances using the R “geosphere” package (Karney, 2013). MMRR was conducted in R (Wang, 2013).

### 2.6 Predictors of morphological divergence

To quantify the relative role of environmental differences and gene flow in phenotypic differentiation among populations, I conducted a PERMANOVA to test whether canopy or gene flow predict pairwise differences in morphology across ponds using the R “vegan” package (Oksanen et al., 2016). The model included canopy as a factor and genetic cluster (as determined by STRUCTURE) as predictor variables in addition to ln‐transformed mass as a covariate. The analysis was repeated using the combined morphological distance matrix for each time period (days 18 and 37) and also for each individual trait. *p*‐values were corrected with sequential Bonferroni.

I also conducted a principal components analysis (PCA) to evaluate differences in morphology across canopy types and genetic clusters. Pond averages for each of the five ln‐transformed morphological measurements in addition to ln‐transformed mass were used for the analysis. Principal components (PC) axes that explained >1% of the variation in the data were retained. The PC values were plotted with 95% confidence ellipses to visually evaluate differences among open‐ and closed‐canopy ponds. ANOVA was used to quantify differences in each of the four PC axes for each time period. *p*‐values were corrected with sequential Bonferroni.

## 3 RESULTS

### 3.1 Common garden experiment

After averaging all individuals within tanks, there were significant differences across ponds in all of the morphological traits (repeated‐measures ANOVA: *p *<* *.012; Table 1). Both mass (*p *=* *4.08 *10^−37^) and muscle depth (*p *=* *.004) varied over time (Table 1). None of the morphological traits varied across spatial blocks (Table 1).

**Table 1:** Morphological variation across ponds. Morphological traits were averaged across ~10 individuals in each tank. Predictors of morphological variation included pond, spatial block, and time (day 18 vs. 37) with ln‐transformed mass as a covariate in the model. Each morphological trait was ln‐transformed for linearity, including tail length (TL), tail depth (TD), body length (BL), body depth (BD), and muscle depth (MD)

Trait	Variable	*df*	SumSq	MeanSq	*F* value	*p* value
Mass.tr	Pond	15	2.27	0.15	6.33	4.30E‐07
	Pond:Block	48	0.65	0.01	0.56	.9751
	Pond:Time	16	68.35	4.27	178.5	4.08E‐37
	Residuals	48	1.15	0.02		
TD.tr	Pond	15	23.15	1.54	8.35	8.69E‐09
	Mass.tr	1	876.6	876.6	4743	7.43E‐49
	Pond:Block	48	14.72	0.31	1.66	.04229
	Pond:Time	16	4.91	0.31	1.66	.08998
	Residuals	47	8.69	0.18		
MD.tr	Pond	15	28.01	1.87	2.38	.0119
	Mass.tr	1	1381	1381	1764	6.36E‐39
	Pond:Block	48	38.43	0.8	1.02	.4699
	Pond:Time	16	33.71	2.11	2.69	.004313
	Residuals	47	36.8	0.78		
BD.tr	Pond	15	22.56	1.5	8.72	4.43E‐09
	Mass.tr	1	867.5	867.5	5030	1.90E‐49
	Pond:Block	48	9.32	0.19	1.13	.3426
	Pond:Time	16	4.64	0.29	1.68	.0844
	Residuals	47	8.11	0.17		
TL.tr	Pond	15	38.54	2.57	23.32	8.69E‐17
	Mass.tr	1	893.6	893.6	8110	2.74E‐54
	Pond:Block	48	5.29	0.11	1	.5014
	Pond:Time	16	3.67	0.23	2.08	.02649
	Residuals	47	5.18	0.11		
BL.tr	Pond	15	26.79	1.79	54.33	1.62E‐24
	Mass.tr	1	695.3	695.3	21148	4.92E‐64
	Pond:Block	48	1.66	0.03	1.05	.4317
	Pond:Time	16	0.49	0.03	0.94	.537
	Residuals	47	1.55	0.03		

### 3.2 Genetic structure

**Figure 2:**
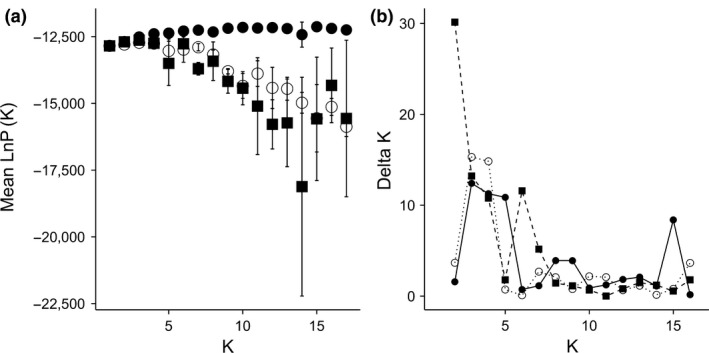
Likelihood and Δ *K* scores for increasing number of genetic clusters. Likelihood (a) and Δ*K* scores (b) for increasing number of clusters (*K*) using either no prior (open circles, dotted line), individual ponds as a prior (closed circles, solid line), pond environment (closed squares, dashed line) as a prior

**Figure 3:**
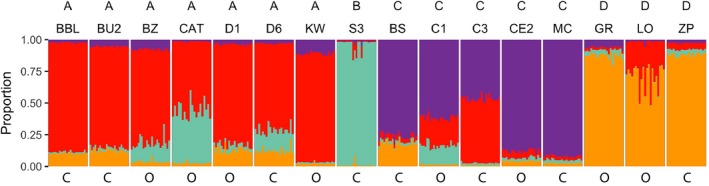
Individual genetic cluster assignments from STRUCTURE analysis. Proportion assigned to each cluster for each individual using pond locations as a prior and assuming *K *=* *4. Pond names above and canopy classification, open (O) and closed (C), below

STRUCTURE analysis indicated a high amount of gene flow among and admixture in most ponds. When run with no prior, Δ*K* peaked at *K *=* *3 and *K *=* *4, with both having the highest likelihood scores (Figure 2a,b). The clusters correspond to geographic locations with ponds grouping with neighboring ponds, with *K *=* *4 identifying more geographic substructuring (Figure 3). With ponds set as the location prior, *K *=* *3 had the highest Δ*K*, with slightly lower peaks at *K *=* *4, *K *=* *5, and *K *=* *15. Likelihood values increased with increasing *K* and leveled off at approximately *K *=* *6 (Figure 2a,b). Again, ponds clustered into geographic locations, with *K *=* *4 identifying geographic substructuring. Although likelihood values increased with increasing *K*, above *K *=* *4 individuals were increasingly split among multiple clusters. Last, when canopy type was used as the location prior, the best‐supported number of clusters was 2 based on both Δ*K* and likelihood values (Figure 2a,b). One of the two clusters contained only one pond (Sullivan 3) and the other cluster contained the remaining 15 ponds. Higher values of *K* showed most individuals being split among different clusters. Further, alpha values did not converge in any run when environment was used as the prior; thus, the results from this analysis should be used with caution. Based on these results we used *K *=* *4 as a conservative estimate of geographic structure. Importantly, there were open‐ and closed‐canopy ponds within each cluster except in the cluster that included only a single pond, Sullivan 3 (Figure 3).

**Figure 4:**
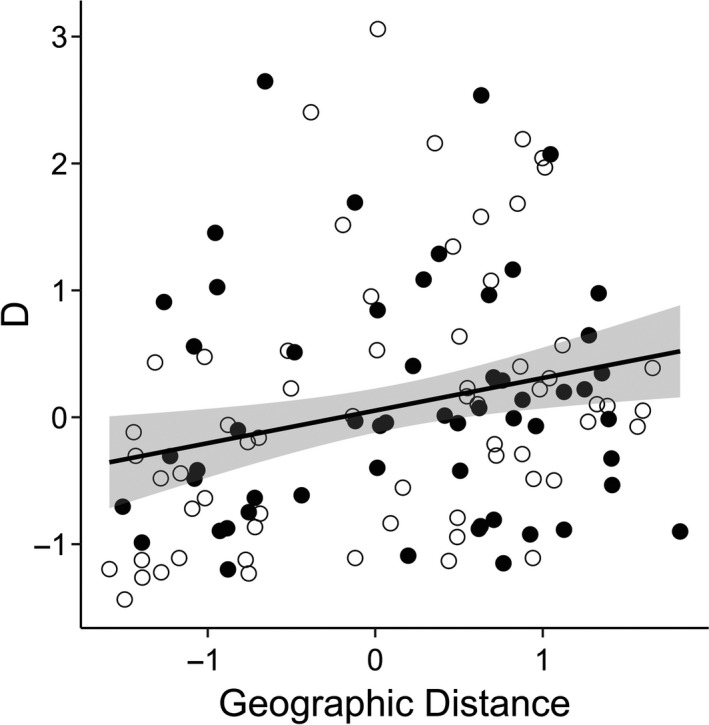
Isolation by distance but not by environment. Gene flow as measured by Jost's *D* increases with greater Euclidean distance between ponds but not by environmental distance. Points represent pairwise comparisons of ponds of similar canopy type (closed circles) or opposite canopy type (open circles). Linear regression line shown with SEM. Both axes centered and standardized

Jost's *D* values indicated there were genetic differences among 54/120 (45%) pairwise pond comparisons (Table S1). In addition, while genetic differentiation, as measured by Jost's *D*, was associated with geographic distance (MMRR: *t *=* *2.71, *p *=* *.03), it was not associated with differences between canopy (*t *=* *0.13, *p *=* *.86; Figure 4; Table S2).

### 3.3 Predictors of Morphological Variation

**Figure 5:**
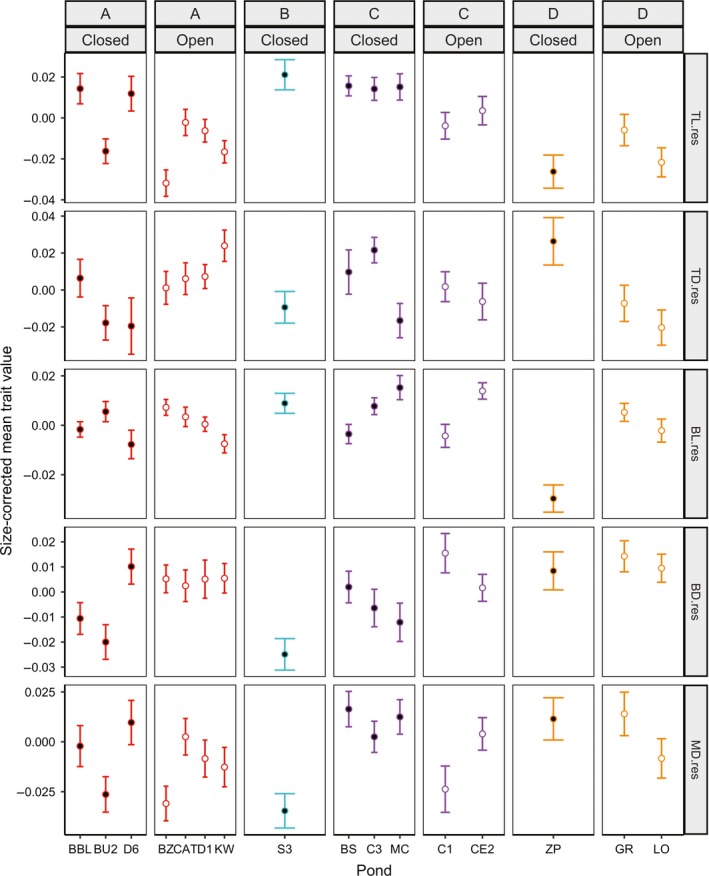
Variation in morphology among ponds at day 18. Individuals within each tank were averaged prior to analysis. Mean size and standard error of the mean were calculated across tanks (*n *= 64). Ponds are categorized as open canopy (open circles) or closed canopy (solid circles). Morphological traits include residuals of ln‐transformed tail length (TL), tail depth (TD), body length (BL), body depth (BD), and muscle depth (MD). Colors correspond to genetic clusters in Figure 3. Differences across canopy types and genetic clusters were analyzed with PERMANOVA with ln‐transformed mass as a covariate

**Figure 6:**
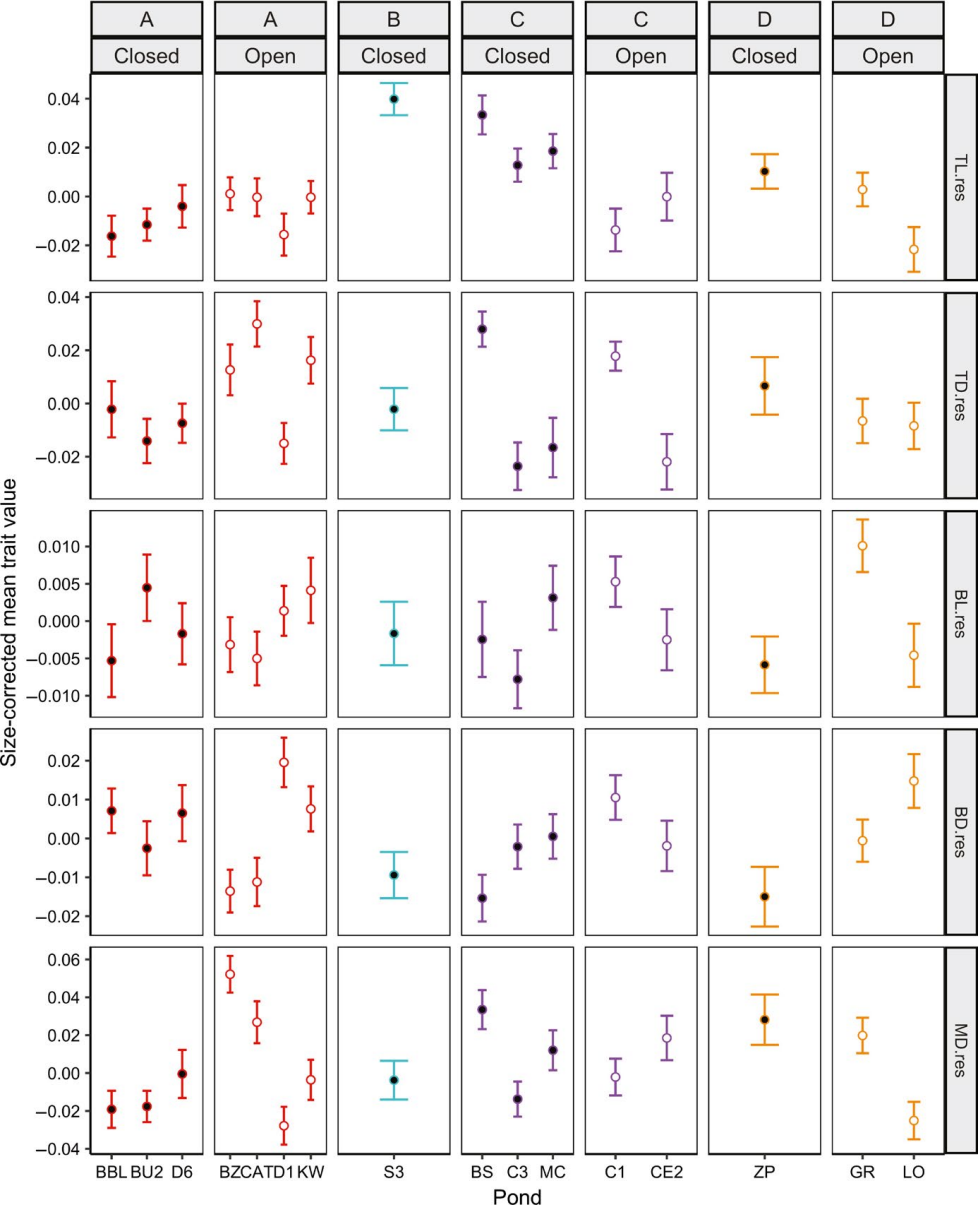
Variation in morphology among ponds at day 37 (Mean, SEM). Individuals within each tank were averaged prior to analysis. Mean size and standard error of the mean were calculated across tanks (*n *= 64). Ponds are categorized as open canopy (open circles) or closed canopy (solid circles). Morphological traits include residuals of ln‐transformed tail length (TL), tail depth (TD), body length (BL), body depth (BD), and muscle depth (MD). Colors correspond to genetic clusters in Figure 3. Differences across canopy types and genetic clusters were analyzed with PERMANOVA with ln‐transformed mass as a covariate

Across all morphological traits combined, the differences in morphology across ponds were significantly different across canopy types (PERMANOVA: *p *<* *.023) at day 18 only but significantly differed across clusters (*p *<* *.001), and was significantly associated with ln‐transformed mass (*p *<* *.001) on both days (Table 2). There were differences among morphological traits in the extent of association with canopy and cluster (Figures 5, 6; Tables S3–S4). At day 18, tail length, body length, and body depth differed across canopy (PERMANOVA: *p *<* *.002, *p *<* *.001, *p *<* *.001, respectively; Table S2). At day 37, only body length significantly differed across canopy types (*p *<* *.001), although tail depth also showed a trend for differences across canopy type (*p *<* *.036; Table S2). All morphological variables aside from muscle depth (*p *>* *.05) differed across clusters and were significantly associated with mass for both time periods (Tables S3–S4).

**Table 2:** Predictors of overall morphological variation with PERMANOVA. Association between overall morphological variation and predictor variables, including canopy, genetic cluster (*K *=* *4). Ln‐transformed mass was included as a covariate in the model. A distance matrix was calculated based on differences in each of the five ln‐transformed morphological traits at days 18 and 37

Time	Variable	*df*	SumSq	MeanSq	*F* value	R^2^	*p* value
18	Canopy	1	32.15	32.15	5.28	0.06	.02298
	Clusters	3	300.4	100.2	16.44	0.57	.000999
	Mass.tr	1	132.8	132.8	21.8	0.25	.000999
	Residuals	10	60.91	6.09		0.12	
	Total	15	526.3			1	
37	Canopy	1	5.26	5.26	1.21	0.03	.3237
	Clusters	3	109	36.32	8.32	0.56	.000999
	Mass.tr	1	37.02	37.02	8.48	0.19	.000999
	Residuals	10	43.67	4.37		0.22	
	Total	15	194.9			1	

Combining morphological traits in a principal components analysis elucidated overall differences across ponds. The PCA returned four PC axes for each time period that each explained at least 1% of the variation in morphology (Tables S5–S6). For day 18, PC1 explained 92% of the variation, loading on all morphological traits equally. PC2 explained 6% of the variation and loaded heavily on a trade‐off between tail length and muscle depth residuals. PC3 explained 1% of the variation and loaded heavily on tail depth residuals versus muscle depth. PC4 explained 1% of the variation and loaded heavily on body depth and tail depth. For day 37, PC1 explained 49% of the variation, loading on all morphological traits equally. PC2 explained 26% of the variation and loaded heavily on a trade‐off between tail length and muscle depth residuals. PC3 explained 14% of the variation and loaded heavily on body depth versus muscle depth. PC4 explained 7% of the variation and loaded heavily on tail depth.

**Figure 7:**
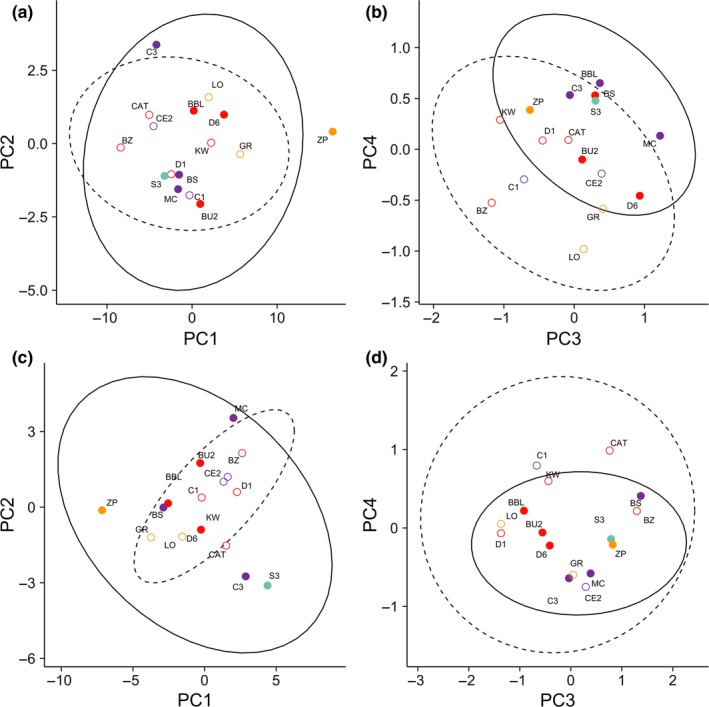
PCA of morphological variation by environmental differences. The PCA included the ln‐transformed values of the five morphological measurements in addition to mass at days 18 (a,b) 37 (c,d) of larval development. Environmental differences plotted as open canopy (open circles) versus closed canopy (filled circles). Genetic clusters shown in colors based on best‐supported STRUCTURE run, *K *=* *4, as shown in Figure 3. PC1 significantly differed across genetic clusters at both time periods (ANOVA: *p *=* *.007, *p *=* *.002, respectively). PC3 and PC4 showed a trend for variation across canopy (ANOVA: *p *=* *.077, *p *=* *.024, respectively) at day 18 only

For both time periods, PC1 significantly differed across genetic clusters (day 18: *p *=* *.007, day 37: *p *=* *.002; Table 3; Figure 7). PC2 showed no variation in association with canopy or genetic cluster (*p *>* *.05; Table 3). PC3 and PC4 showed a trend toward significant differences across canopy type at day 18 (*p *=* *.077, *p *=* *.024), although was not significant following sequential Bonferroni correction (Table 3; Figure 7b).

**Table 3:** Predictors of overall morphological variation with PCA. Association between overall morphological variation and predictor variables, including canopy, genetic cluster (*K *=* *4) for days 18 and 37 were assessed with ANOVA

Time	PC Axis	Variable	*df*	SumSq	MeanSq	*F* value	*p* value
18	PC1	Canopy	1	29.42	29.42	2.053	.180
		Cluster	3	297.51	99.17	6.921	.007
		Residuals	11	157.62	14.33		
	PC2	Canopy	1	0.00	0.00	0.001	.972
		Cluster	3	2.40	0.80	0.308	.819
		Residuals	11	28.54	2.59		
	PC3	Canopy	1	1.61	1.61	3.805	.077
		Cluster	3	0.35	0.12	0.275	.842
		Residuals	11	4.66	0.42		
	PC4	Canopy	1	1.16	1.16	6.850	.024
		Cluster	3	0.40	0.13	0.790	.524
		Residuals	11	1.87	0.17		
37	PC1	Canopy	1	3.71	3.71	1.135	.309
		Cluster	3	93.23	31.08	9.522	.002
		Residuals	11	35.90	3.26		
	PC2	Canopy	1	0.52	0.52	0.181	.679
		Cluster	3	14.02	4.67	1.618	.241
		Residuals	11	31.77	2.89		
	PC3	Canopy	1	0.52	0.52	0.590	.459
		Cluster	3	1.07	0.36	0.402	.754
		Residuals	11	9.79	0.89		
	PC4	Canopy	1	0.38	0.38	1.408	.260
		Cluster	3	0.68	0.23	0.843	.498
		Residuals	11	2.96	0.27		

## 4 DISCUSSION

The presence of fine‐scale phenotypic variation in wood frog populations has been hypothesized to be due to either population isolation or due to very strong selection within ponds (Relyea, 2002b). Determining the relative contribution of selection and isolation to population divergence is important for understanding fine‐scale evolutionary processes and ultimately speciation (Ohmer, Robertson, & Zamudio, 2009). Using a common garden experiment, I demonstrated that larval wood frog populations exhibit significant morphological variation across an environmental gradient of canopy cover (Figure 5; Table 2), consistent with previous research (Relyea, 2002b, 2005). Wood frog tadpoles from different ponds showed significant morphological variation associated with canopy type with a trade‐off between tail length and body depth (Figure 5). However, contrary to expectations, there was no evidence that gene flow is limited across the environmental gradient (Figures 3, 4). The morphological differences occur over surprisingly small geographic scales, with gene flow between neighboring ponds of opposite canopy type (Figure 1).

While there was some genetic population structure across the entire study area, there was evidence of high levels of admixture among ponds (Figure 3). Genetic clusters corresponded to location rather than canopy types (Figures 2, 3), with each genetic cluster containing both open‐ and closed‐canopy ponds (excluding the cluster that contained only a single pond, Sullivan 3). Genetic analysis confirms that dispersal and gene flow likely occurs across nearby ponds and even across canopy types (Figures 2, 3). Further, I found no evidence that dispersal or gene flow was limited by environmental differences among ponds. Although genetic divergence increased with geographic distance, it was not associated with canopy type (Figure 4; Table S2). As a result, there is discordance among genetic and morphological variation among larval wood frog populations. This discordance is strongest during the selective period, when larvae are still small enough to be vulnerable to many aquatic predators (day 18), but dissipates when larvae are larger (day 37), with morphological variation becoming more closely associated with genetic divergence instead of environmental differences (Table 2). Taken together, the genetic and morphological results support the hypothesis that for some traits, selection is sufficiently strong within local environments so as to counteract gene flow that occurs.

### 4.1 Variation across traits

When considering individual traits, there was variation in the extent to which each trait diverged across pond types or with gene flow. There are a number of differences among the traits that may explain the varying patterns of phenotypic divergence, including differences in selection on each trait, the type of trait, the degree of plasticity, the amount of heritability or heritable plasticity, and correlations among traits. Interestingly, the results fit the *a priori* expectations derived from previous research on heritabilities of and selection on each trait (Relyea, 2005; Van Buskirk & Relyea, 1998). During the time when tadpoles are more susceptible to aquatic gape‐limited predators (day 18), traits which are heritable and under selection by predators (tail length and body depth: Van Buskirk & Relyea, 1998; Relyea, 2005) showed variation due to environment (Tables 2, 3; Table S2), whereas traits that have been reported to have heritable plasticity, including tail depth (Relyea, 2005), or heritable (Relyea, 2005) but with little evidence of being under selection by *Anax* predators, including muscle depth (Van Buskirk & Relyea, 1998), showed little divergence across environment (Tables 2, 3; Table S2). While the effects of trait differences were not explicitly assessed in this study, the results presented here in combination with results from previous studies (e.g., Relyea, 2001a, 2005; Van Buskirk & Relyea, 1998) provide a number of avenues for future research, particularly with regard to the role of these trait differences in generating variation in the effect of gene flow on local adaptation. Although other environmental differences along this gradient could be contributing to the phenotypic differences among populations, the correspondence between the observed patterns of phenotypic variation and the *a priori* predictions as to which traits should show a pattern of divergence among canopy types lends support to the hypothesis that these morphological traits are associated with selective differences in the environment.

The variation in effects of selection and gene flow on different traits implies that multiple processes may be occurring simultaneously across the genome to generate phenotypic divergence among populations. This result is consistent with studies that have demonstrated variation in the impacts of gene flow on different parts of the genome (e.g., Nosil et al., 2008) as well as variation in phenotypic traits. For example across geographic clines, there is frequently variation in the extent to which various genes and phenotypic traits show introgression (Baldassarre, White, Karubian, & Webster, 2014). This variation across traits may explain why studies of gene flow and local adaptation have produced such divergent results, as studies focusing on different traits or different suites of traits may find conflicting results depending on the relative roles of gene flow and selection on each. For species in which plasticity of some traits is under selection, such as the wood frog (Relyea, 2005), these processes may be even more difficult to disentangle. Gene flow may even facilitate the evolution of heritable plasticity by providing variation upon which selection can act (Crispo, 2008; Rasanen & Hendry, 2008). Further theoretical models and empirical examples are needed to fully understand how different phenotypic traits respond to gene flow and selection.

### 4.2 Temporal patterns

Although both selection and gene flow were associated with some of the variation across traits during both time periods, these patterns were not always constant over time. At day 18, there was significant variation in morphological differences associated with environmental differences among ponds; however, this difference dissipated by day 37 (Tables S3–S4). In comparison, there was significant variation in phenotype associated with gene flow during both time periods. The only trait that continued to be associated with canopy across both time periods was body length, while the associations with both tail length and body depth dissipated (Tables S3–S4). What these results suggest is that the effects of both selection and gene flow may vary through larval development, demonstrating the importance of measuring phenotypic divergence over multiple time points and at times that are relevant to the processes being evaluated. Many studies assessing the effects of selection and gene flow on local adaptation have primarily focused on measuring traits at a single time point (e.g., Smith et al., 1997; Storfer, Cross, Rush, & Caruso, 1999; Nosil & Crespi, 2004). By incorporating temporal samples into these types of studies, we will have a better opportunity to understand the significance of phenotypic variation in the face of gene flow and may be able to uncover the occurrence of additional processes that otherwise would have been missed. For example, for some traits, the effects of selection may be compensated for over time (e.g., body depth), whereas for others (e.g., tail length) the effects of selection may be longer lasting. Such longer lasting effects may indicate potential for carry‐over effects, with environmental conditions in the larval stage impacting traits in adults (Denver & Maher, 2010).

### 4.3 Spatial patterns

Interestingly, in addition to differences across canopy types, there was also some unexpected evidence that morphological variation is associated with geographic genetic structure across the four geographic regions identified by the clustering analysis. Specifically, differences in overall morphology (Tables 2, 3) and multiple individual morphological measures (Tables S3–S4) varied in association with geographically based genetic clusters. The principal components plots, while not significant, suggest that within clusters there is a trend toward morphological differences between open‐ and closed‐canopy ponds; however, there is variation among clusters in the direction of those differences (Figure 7). This variation in the extent and direction of morphological differences between open‐ and closed‐canopy ponds among different genetic clusters could be due to drift or founder events. Alternatively, these results could suggest that environmental differences between open‐ and closed‐canopy ponds may vary across the study area. This pattern could arise due to larger‐scale environmental variation across the study region, for instance if predator levels or composition varied across space, thus imposing different selection pressures. In fact, wood frogs do show variation in both morphological and behavioral responses to different predators (Relyea, 2001a). In addition to variation in predators (Relyea, 2002b), many other environmental variables are correlated with the open‐ and closed‐canopy gradient, such as resource availability, dissolved oxygen, hydroperiod, warmth (Werner & Glennemeier, 1999), or competition (Werner et al., 2007). Further research should investigate variation in selective pressures due to environmental differences that may occur at these larger spatial scales. The variation in morphology across space illustrates the importance in taking into account both environmental variation and gene flow, as differences among open‐ and closed‐canopy ponds could be obscured by this additional variation.

### 4.4 Alternative hypotheses

Two alternative hypotheses could explain the observed phenotypic differences among canopy types, including early environmental cues or maternal effects; however, there is little evidence to support either of these hypotheses. First, the phenotypic differences could be due to exposure to cues (e.g., predator chemical cues) in the ponds during the approximately 24 hours before eggs were collected. However, recent research suggests that for larval wood frogs, such cues must be associated with actual costs (e.g., chemical cues from depredated conspecifics) in order for predator‐related morphologies and behaviors to be induced (Ferrari & Chivers, 2009). This situation is not likely in this system because all eggs are laid simultaneously; thus, no conspecific larvae would have been present for predators to feed on when eggs were deposited. Similarly, the pattern of divergence among open‐ and closed‐canopy ponds does not appear to be due to maternal effects, because if maternal effects were responsible, then we would expect higher phenotypic variance among individuals in high rather than low connectivity populations (i.e., traits would be bimodally distributed), since maternal effects should not be affected by gene flow. However, there was no evidence of bimodal distributions in highly connected populations. Moreover, differences in hatchling size among populations, an important potential maternal effect (Urban, 2007), were not correlated with environment (*r *=* *−.025, *p *=* *.554). Further evidence that the effects are not due to early environmental cues or maternal effects is that traits that are known to have high levels of heritable plasticity (e.g., body length and tail depth) showed no variation due to the selective regime. While these results provide some evidence to suggest that neither early environmental cues nor maternal effects are responsible for the observed pattern, more research will be needed to assess the relative contribution of these processes to phenotypic differences among populations. Isolating maternal effects would require raising wood frogs in the laboratory over multiple generations. This approach remains a challenge for longer‐lived organisms that require at least a year before sexual maturity.

While there is increasing evidence that divergence is possible with gene flow (Emelianov et al., 2004; Niemiller et al., 2008; Smith et al., 1997), most studies assessing the effects of gene flow on local adaptation have found increasing phenotypic divergence with decreasing gene flow or population connectivity (see Rasanen & Hendry, 2008 and references therein). There is increasing evidence for gene flow among populations experiencing opposing selection pressures (Crispo, Bentzen, Reznick, Kinnison, & Hendry, 2006; Crispo & Chapman, 2008; Richter‐Boix, Teplitsky, Rogell, & Laurila, 2010). Theory predicts this pattern when increasing immigration of maladapted individuals into a population increases the strength of selection within populations due to a “migration load” (Bolnick & Nosil, 2007). As a result, there may be no net change in trait frequencies across time despite immigration (Bolnick & Nosil, 2007). This mechanism has been proposed to explain trait means within isolated and connected *Timema* walking‐stick populations (Bolnick & Nosil, 2007) and could additionally explain why there is little effect of gene flow on divergence of these wood frog populations across open‐ and closed‐canopy ponds. Future research assessing differences in selection differentials and fitness within more isolated and connected populations will be necessary to determine if this mechanism is responsible for the observed patterns of divergence.

## 5 CONCLUSION

Previous research on the effects of gene flow on local adaptation has provided mixed results, with support for gene flow as a constraining force (Bridle & Vines, 2007; Kawecki & Ebert, 2004; Lenormand, 2002; Slatkin, 1985), a facilitating force (Bridle & Vines, 2007), or alternatively having little influence on divergence of populations (Emelianov et al., 2004; Niemiller et al., 2008; Smith et al., 1997). The association between morphological variation in larval wood frog populations and environment within genetic clusters suggests that local adaptation may be occurring in the face of gene flow. This study adds to the mounting evidence that even over small spatial scales selection may be strong enough to overpower the homogenizing effects of gene flow (Emelianov et al., 2004; Niemiller et al., 2008; Smith et al., 1997). While overall there was a general pattern of phenotypic divergence incongruent with gene flow, there were also differences across stages of larval development in the extent to which traits showed divergence. Future research should focus on understanding the mechanisms allowing for divergence with gene flow and evaluating the consequences for individual and population fitness.

## CONFLICT OF INTEREST

None declared.
