# Supplementary material for: Microgeographic morphological variation across larval wood frog populations associated with environment despite gene flow
Source: Ecol Evol. 2018 Feb 1;8(5):2504–17. doi: 10.1002/ece3.3829 (PMC5838061; doi:10.1002/ece3.3829)
Supplement: Supplementary file 1 [file ECE3-8-2504-s001.docx]

**SUPPLEMENTARY FILES**

**Supplementary Table 1. Jost’s D** The observed Jost’s *D* value was calculated for all pairwise pond comparisons. The bias corrected lower and upper 95% CI were calculated with 1000 bootstraps. Pairwise Jost’s *D* values with CI that do not overlap 0 are indicated with an asterisk. For each pairwise comparison, the pond abbreviation, assigned genetic cluster (Clust), and canopy type (Can) are shown for comparison.

| Pond1 | Clust1 | Can1 | Pond2 | Clust2 | Can2 | Observed | Lower CI | Upper CI | * |
| --- | --- | --- | --- | --- | --- | --- | --- | --- | --- |
| D6 | A | Closed | MC | C | Closed | 0.0541 | 0.009 | 0.1095 | * |
| BZ | A | Open | MC | C | Closed | 0.0404 | -0.0136 | 0.1043 |  |
| C3 | C | Closed | MC | C | Closed | 0.0325 | -0.014 | 0.0865 |  |
| GR | D | Open | MC | C | Closed | 0.0574 | 0.0058 | 0.1246 | * |
| BBL | A | Closed | MC | C | Closed | 0.0631 | -9e-04 | 0.1364 |  |
| BS | C | Closed | MC | C | Closed | 0.093 | 0.0301 | 0.1737 | * |
| C1 | C | Open | MC | C | Closed | 0.0343 | -0.0102 | 0.0898 |  |
| CAT | A | Open | MC | C | Closed | 0.0607 | 0.0081 | 0.1346 | * |
| D1 | A | Open | MC | C | Closed | 0.0669 | 0.0105 | 0.1371 | * |
| BU2 | A | Closed | MC | C | Closed | 0.0529 | 0.0037 | 0.1135 | * |
| CE2 | C | Open | MC | C | Closed | 0.0326 | -0.013 | 0.0951 |  |
| KW | A | Open | MC | C | Closed | 0.0319 | -0.013 | 0.0899 |  |
| LO | D | Open | MC | C | Closed | 0.1488 | 0.0638 | 0.2449 | * |
| C1 | C | Open | D1 | A | Open | 0.0352 | -0.0198 | 0.1064 |  |
| BBL | A | Closed | D1 | A | Open | -0.0089 | -0.0395 | 0.0469 |  |
| C3 | C | Closed | D1 | A | Open | 0.0125 | -0.0325 | 0.0709 |  |
| BZ | A | Open | D1 | A | Open | 0.0106 | -0.0304 | 0.0662 |  |
| BS | C | Closed | D1 | A | Open | 0.1267 | 0.0614 | 0.1999 | * |
| D6 | A | Closed | D1 | A | Open | 0.0222 | -0.0232 | 0.0865 |  |
| BU2 | A | Closed | D1 | A | Open | 0.0047 | -0.0382 | 0.0605 |  |
| CE2 | C | Open | D1 | A | Open | 0.0622 | 0.0065 | 0.13 | * |
| CAT | A | Open | D1 | A | Open | 0.0402 | -0.0161 | 0.1162 |  |
| BZ | A | Open | C3 | C | Closed | 0.0294 | -0.0138 | 0.0824 |  |
| BBL | A | Closed | C3 | C | Closed | 0.0184 | -0.0314 | 0.0871 |  |
| BS | C | Closed | C3 | C | Closed | 0.0981 | 0.0276 | 0.1791 | * |
| BU2 | A | Closed | C3 | C | Closed | 0.0035 | -0.0345 | 0.0519 |  |
| C1 | C | Open | C3 | C | Closed | 4e-04 | -0.0317 | 0.0457 |  |
| BS | C | Closed | S3 | B | Closed | 0.1686 | 0.103 | 0.2412 | * |
| BBL | A | Closed | S3 | B | Closed | 0.0902 | 0.0424 | 0.1548 | * |
| KW | A | Open | S3 | B | Closed | 0.0949 | 0.0536 | 0.1383 | * |
| CAT | A | Open | S3 | B | Closed | 0.0205 | -0.008 | 0.0569 |  |
| BZ | A | Open | S3 | B | Closed | 0.0634 | 0.0217 | 0.1155 | * |
| D1 | A | Open | S3 | B | Closed | 0.1194 | 0.0597 | 0.1916 | * |
| MC | C | Closed | S3 | B | Closed | 0.0758 | 0.0322 | 0.1299 | * |
| CE2 | C | Open | S3 | B | Closed | 0.158 | 0.0903 | 0.2374 | * |
| D6 | A | Closed | S3 | B | Closed | 0.0491 | 0.0061 | 0.1063 | * |
| BU2 | A | Closed | S3 | B | Closed | 0.1007 | 0.0549 | 0.1511 | * |
| C1 | C | Open | S3 | B | Closed | 0.0742 | 0.0251 | 0.1293 | * |
| C3 | C | Closed | S3 | B | Closed | 0.1167 | 0.0716 | 0.1655 | * |
| GR | D | Open | S3 | B | Closed | 0.1222 | 0.0639 | 0.1934 | * |
| LO | D | Open | S3 | B | Closed | 0.1865 | 0.1127 | 0.2778 | * |
| BBL | A | Closed | CAT | A | Open | 0.0258 | -0.0228 | 0.0907 |  |
| C1 | C | Open | CAT | A | Open | 0.0362 | -0.0084 | 0.0939 |  |
| BS | C | Closed | CAT | A | Open | 0.1474 | 0.0801 | 0.221 | * |
| BZ | A | Open | CAT | A | Open | 0.0229 | -0.02 | 0.078 |  |
| BU2 | A | Closed | CAT | A | Open | 0.0763 | 0.0251 | 0.1351 | * |
| C3 | C | Closed | CAT | A | Open | 0.0765 | 0.0243 | 0.1382 | * |
| CAT | A | Open | D6 | A | Closed | 0.0015 | -0.0301 | 0.057 |  |
| CE2 | C | Open | D6 | A | Closed | 0.0812 | 0.0142 | 0.1618 | * |
| C1 | C | Open | D6 | A | Closed | 0.0053 | -0.0327 | 0.0558 |  |
| BBL | A | Closed | D6 | A | Closed | 0.0155 | -0.0276 | 0.0742 |  |
| C3 | C | Closed | D6 | A | Closed | 0.0522 | 0.0068 | 0.1104 | * |
| BZ | A | Open | D6 | A | Closed | -0.0014 | -0.0267 | 0.0447 |  |
| BS | C | Closed | D6 | A | Closed | 0.0711 | 0.0116 | 0.1378 | * |
| BU2 | A | Closed | D6 | A | Closed | 0.0268 | -0.0187 | 0.0865 |  |
| BBL | A | Closed | BZ | A | Open | 0.0053 | -0.036 | 0.0667 |  |
| D1 | A | Open | ZP | D | Closed | 0.0409 | -0.02 | 0.1239 |  |
| BZ | A | Open | ZP | D | Closed | 0.0709 | 0.0126 | 0.1414 | * |
| MC | C | Closed | ZP | D | Closed | 0.0394 | -0.0086 | 0.0981 |  |
| KW | A | Open | ZP | D | Closed | 0.0704 | 0.0032 | 0.1479 | * |
| GR | D | Open | ZP | D | Closed | 0.0403 | -0.0285 | 0.1227 |  |
| S3 | B | Closed | ZP | D | Closed | 0.0661 | 0.011 | 0.146 | * |
| CAT | A | Open | ZP | D | Closed | 0.0663 | 0.0105 | 0.1434 | * |
| BBL | A | Closed | ZP | D | Closed | 0.0595 | 8e-04 | 0.1395 | * |
| BS | C | Closed | ZP | D | Closed | 0.0505 | -0.0082 | 0.124 |  |
| C3 | C | Closed | ZP | D | Closed | 0.0686 | 0.0039 | 0.1455 | * |
| C1 | C | Open | ZP | D | Closed | 0.0631 | 6e-04 | 0.133 | * |
| BU2 | A | Closed | ZP | D | Closed | 0.0144 | -0.0396 | 0.0819 |  |
| LO | D | Open | ZP | D | Closed | 0.0509 | -0.0119 | 0.139 |  |
| D6 | A | Closed | ZP | D | Closed | 0.0672 | 0.0074 | 0.1381 | * |
| CE2 | C | Open | ZP | D | Closed | 0.0782 | 0.0102 | 0.1552 | * |
| BBL | A | Closed | LO | D | Open | 0.0172 | -0.0398 | 0.0901 |  |
| BS | C | Closed | LO | D | Open | 0.112 | 0.0515 | 0.1855 | * |
| D6 | A | Closed | LO | D | Open | 0.0539 | -0.0085 | 0.1347 |  |
| D1 | A | Open | LO | D | Open | 0.0517 | -0.0169 | 0.1426 |  |
| BU2 | A | Closed | LO | D | Open | 0.1423 | 0.0713 | 0.2204 | * |
| C3 | C | Closed | LO | D | Open | 0.1003 | 0.0263 | 0.1866 | * |
| KW | A | Open | LO | D | Open | 0.1041 | 0.0376 | 0.1865 | * |
| BZ | A | Open | LO | D | Open | 0.0506 | -0.0115 | 0.1269 |  |
| CAT | A | Open | LO | D | Open | 0.1271 | 0.05 | 0.2152 | * |
| GR | D | Open | LO | D | Open | 0.0778 | 0.003 | 0.1652 | * |
| C1 | C | Open | LO | D | Open | 0.1095 | 0.0276 | 0.1979 | * |
| CE2 | C | Open | LO | D | Open | 0.0954 | 0.0224 | 0.1857 | * |
| D1 | A | Open | GR | D | Open | 0.0567 | -0.0046 | 0.1305 |  |
| C1 | C | Open | GR | D | Open | 0.0134 | -0.0361 | 0.0851 |  |
| BS | C | Closed | GR | D | Open | 0.0324 | -0.0227 | 0.1078 |  |
| CE2 | C | Open | GR | D | Open | 0.015 | -0.0476 | 0.0964 |  |
| BU2 | A | Closed | GR | D | Open | 0.0503 | -0.0119 | 0.1216 |  |
| C3 | C | Closed | GR | D | Open | 0.052 | -0.004 | 0.1188 |  |
| BZ | A | Open | GR | D | Open | 0.0162 | -0.0332 | 0.0849 |  |
| BBL | A | Closed | GR | D | Open | 0.058 | -0.0141 | 0.1478 |  |
| CAT | A | Open | GR | D | Open | 0.0515 | 0 | 0.1109 |  |
| D6 | A | Closed | GR | D | Open | 0.0191 | -0.03 | 0.0832 |  |
| D6 | A | Closed | KW | A | Open | 0.0465 | -0.0034 | 0.1125 |  |
| D1 | A | Open | KW | A | Open | 0.0014 | -0.0406 | 0.0638 |  |
| BBL | A | Closed | KW | A | Open | 0 | -0.0443 | 0.0672 |  |
| BS | C | Closed | KW | A | Open | 0.1391 | 0.068 | 0.2207 | * |
| BU2 | A | Closed | KW | A | Open | 0.0046 | -0.0354 | 0.0598 |  |
| GR | D | Open | KW | A | Open | 0.0303 | -0.0319 | 0.1121 |  |
| BZ | A | Open | KW | A | Open | 0.0146 | -0.0277 | 0.0678 |  |
| C1 | C | Open | KW | A | Open | 0.0153 | -0.0229 | 0.0599 |  |
| C3 | C | Closed | KW | A | Open | 0.0043 | -0.0287 | 0.0492 |  |
| CAT | A | Open | KW | A | Open | 0.021 | -0.0264 | 0.0839 |  |
| CE2 | C | Open | KW | A | Open | 0.0816 | 0.0169 | 0.1601 | * |
| BZ | A | Open | BU2 | A | Closed | 0.0159 | -0.0246 | 0.072 |  |
| BS | C | Closed | BU2 | A | Closed | 0.096 | 0.0318 | 0.1774 | * |
| BBL | A | Closed | BU2 | A | Closed | 0.0259 | -0.0203 | 0.09 |  |
| BBL | A | Closed | BS | C | Closed | 0.1436 | 0.0766 | 0.2268 | * |
| BZ | A | Open | BS | C | Closed | 0.0635 | 0.0102 | 0.1332 | * |
| BBL | A | Closed | C1 | C | Open | 0.0443 | -0.0109 | 0.1157 |  |
| BS | C | Closed | C1 | C | Open | 0.0723 | 0.013 | 0.1461 | * |
| BU2 | A | Closed | C1 | C | Open | 0.0053 | -0.0324 | 0.0596 |  |
| BZ | A | Open | C1 | C | Open | 0.0061 | -0.0362 | 0.058 |  |
| CAT | A | Open | CE2 | C | Open | 0.1638 | 0.0773 | 0.2577 | * |
| C1 | C | Open | CE2 | C | Open | 0.0354 | -0.0237 | 0.1218 |  |
| BZ | A | Open | CE2 | C | Open | 0.0532 | -0.0026 | 0.1249 |  |
| C3 | C | Closed | CE2 | C | Open | 0.045 | -0.0084 | 0.1134 |  |
| BS | C | Closed | CE2 | C | Open | 0.0484 | -0.0121 | 0.1252 |  |
| BBL | A | Closed | CE2 | C | Open | 0.058 | -0.0021 | 0.1314 |  |
| BU2 | A | Closed | CE2 | C | Open | 0.0558 | 5e-04 | 0.1302 | * |

**Supplementary Table 2. Isolation by Environment.** Geographic distance but not environmental distance predicts genetic divergence among ponds. Genetic divergence measured using Jost’s *D*. Geographic distance measured as Haversine distance. Environmental distance measured as Euclidean difference in canopy cover. Each variable was centered and standardized prior to analysis.

| Variable | Coefficients | TValue | T_PValue | R2 | FValue | F_PValue |
| --- | --- | --- | --- | --- | --- | --- |
| Intercept | 0.04 | 0.31 | 0.8014 | 0.06 | 3.67 | 1e-04 |
| Geographic Dist | 0.26 | 2.71 | 0.0301 |  |  |  |
| Environmental Dist | 0.02 | 0.13 | 0.8625 |  |  |  |

**Supplementary Table 3. Predictors of individual trait variation at day 18 with PERMANOVA.** Association between individual trait variation at day 18 and predictor variables, including canopy and genetic cluster (*K*=4). A distance matrix was calculated for each morphological trait based on the ln-transformed values.

| Trait | Variable | DF | SumSq | MeanSq | FValue | R2 | PValue |
| --- | --- | --- | --- | --- | --- | --- | --- |
| Mass.tr | Canopy | 1 | 0.06 | 0.06 | 3.73 | 0.11 | 0.06494 |
|  | Clusters | 3 | 0.32 | 0.11 | 6.22 | 0.56 | 0.04895 |
|  | Residuals | 11 | 0.19 | 0.02 |  | 0.33 |  |
|  | Total | 15 | 0.58 |  |  | 1 |  |
| TD.tr | Canopy | 1 | 6.07 | 6.07 | 5.43 | 0.07 | 0.04096 |
|  | Clusters | 3 | 51.96 | 17.32 | 15.5 | 0.61 | 0.001998 |
|  | Mass.tr | 1 | 16.61 | 16.61 | 14.86 | 0.19 | 0.002997 |
|  | Residuals | 10 | 11.18 | 1.12 |  | 0.13 |  |
|  | Total | 15 | 85.83 |  |  | 1 |  |
| MD.tr | Canopy | 1 | 2.25 | 2.25 | 0.59 | 0.02 | 0.4835 |
|  | Clusters | 3 | 42.98 | 14.33 | 3.78 | 0.4 | 0.07393 |
|  | Mass.tr | 1 | 24.53 | 24.53 | 6.47 | 0.23 | 0.02697 |
|  | Residuals | 10 | 37.92 | 3.79 |  | 0.35 |  |
|  | Total | 15 | 107.7 |  |  | 1 |  |
| BD.tr | Canopy | 1 | 11.22 | 11.22 | 13.69 | 0.14 | 0.004995 |
|  | Clusters | 3 | 39.31 | 13.1 | 15.99 | 0.5 | 0.000999 |
|  | Mass.tr | 1 | 19.24 | 19.24 | 23.48 | 0.25 | 0.000999 |
|  | Residuals | 10 | 8.19 | 0.82 |  | 0.11 |  |
|  | Total | 15 | 77.97 |  |  | 1 |  |
| TL.tr | Canopy | 1 | 4.69 | 4.69 | 20.87 | 0.03 | 0.000999 |
|  | Clusters | 3 | 101.6 | 33.87 | 150.8 | 0.7 | 0.000999 |
|  | Mass.tr | 1 | 36.53 | 36.53 | 162.7 | 0.25 | 0.000999 |
|  | Residuals | 10 | 2.25 | 0.22 |  | 0.02 |  |
|  | Total | 15 | 145.1 |  |  | 1 |  |
| BL.tr | Canopy | 1 | 7.93 | 7.93 | 57.83 | 0.07 | 0.000999 |
|  | Clusters | 3 | 64.57 | 21.52 | 157 | 0.59 | 0.000999 |
|  | Mass.tr | 1 | 35.89 | 35.89 | 261.8 | 0.33 | 0.000999 |
|  | Residuals | 10 | 1.37 | 0.14 |  | 0.01 |  |
|  | Total | 15 | 109.8 |  |  | 1 |  |

**Supplementary Table 4. Predictors of individual trait variation at day 37 with PERMANOVA.** Association between individual trait variation at day 37 and predictor variables, including canopy and genetic cluster (*K*=4). A distance matrix was calculated for each morphological trait based on the ln-transformed values.

| Trait | Variable | DF | SumSq | MeanSq | FValue | R2 | PValue |
| --- | --- | --- | --- | --- | --- | --- | --- |
| Mass.tr | Canopy | 1 | 0 | 0 | 0.72 | 0.03 | 0.4256 |
|  | Clusters | 3 | 0.08 | 0.03 | 4.88 | 0.56 | 0.01399 |
|  | Residuals | 11 | 0.06 | 0.01 |  | 0.42 |  |
|  | Total | 15 | 0.14 |  |  | 1 |  |
| TD.tr | Canopy | 1 | 1.7 | 1.7 | 6.71 | 0.07 | 0.03097 |
|  | Clusters | 3 | 17.97 | 5.99 | 23.66 | 0.75 | 0.000999 |
|  | Mass.tr | 1 | 1.65 | 1.65 | 6.52 | 0.07 | 0.02697 |
|  | Residuals | 10 | 2.53 | 0.25 |  | 0.11 |  |
|  | Total | 15 | 23.86 |  |  | 1 |  |
| MD.tr | Canopy | 1 | 0.77 | 0.77 | 0.31 | 0.01 | 0.5594 |
|  | Clusters | 3 | 25.44 | 8.48 | 3.41 | 0.49 | 0.04895 |
|  | Mass.tr | 1 | 0.79 | 0.79 | 0.32 | 0.02 | 0.5844 |
|  | Residuals | 10 | 24.89 | 2.49 |  | 0.48 |  |
|  | Total | 15 | 51.89 |  |  | 1 |  |
| BD.tr | Canopy | 1 | 1.49 | 1.49 | 2.79 | 0.04 | 0.1219 |
|  | Clusters | 3 | 15.28 | 5.09 | 9.58 | 0.45 | 0.005994 |
|  | Mass.tr | 1 | 11.71 | 11.71 | 22.01 | 0.35 | 0.001998 |
|  | Residuals | 10 | 5.32 | 0.53 |  | 0.16 |  |
|  | Total | 15 | 33.8 |  |  | 1 |  |
| TL.tr | Canopy | 1 | 0 | 0 | 0 | 0 | 0.967 |
|  | Clusters | 3 | 35.77 | 11.92 | 11.22 | 0.6 | 0.000999 |
|  | Mass.tr | 1 | 13.19 | 13.19 | 12.41 | 0.22 | 0.005994 |
|  | Residuals | 10 | 10.63 | 1.06 |  | 0.18 |  |
|  | Total | 15 | 59.6 |  |  | 1 |  |
| BL.tr | Canopy | 1 | 1.3 | 1.3 | 42.81 | 0.05 | 0.000999 |
|  | Clusters | 3 | 14.5 | 4.83 | 158.8 | 0.56 | 0.000999 |
|  | Mass.tr | 1 | 9.68 | 9.68 | 318.1 | 0.38 | 0.000999 |
|  | Residuals | 10 | 0.3 | 0.03 |  | 0.01 |  |
|  | Total | 15 | 25.79 |  |  | 1 |  |

**Supplementary Table 5. PCA Results**  Proportion of variation explained by each principal component axis with loadings for each morphological trait.

| Trait | PC1 | PC2 | PC3 | PC4 | PC5 | PC6 |
| --- | --- | --- | --- | --- | --- | --- |
| Proportion of Variance | 0.92 | 0.06 | 0.01 | 0.01 | 0 | 0 |
| TD.tr | -0.41 | 0.15 | -0.69 | 0.56 | -0.11 | -0.01 |
| MD.tr | -0.43 | 0.73 | 0.52 | 0.12 | -0.05 | 0.02 |
| BD.tr | -0.39 | 0.16 | -0.35 | -0.66 | 0.5 | -0.03 |
| TL.tr | -0.52 | -0.58 | 0.35 | 0.3 | 0.42 | 0 |
| BL.tr | -0.47 | -0.29 | 0.03 | -0.37 | -0.75 | -0.06 |
| Mass.tr | -0.03 | -0.03 | -0.02 | -0.04 | -0.03 | 1 |

**Supplementary Table 6. PCA Results**  Proportion of variation explained by each principal component axis with loadings for each morphological trait.

| Trait | PC1 | PC2 | PC3 | PC4 | PC5 | PC6 |
| --- | --- | --- | --- | --- | --- | --- |
| Proportion of Variance | 0.68 | 0.24 | 0.06 | 0.02 | 0 | 0 |
| TD.tr | 0.39 | 0.07 | 0.04 | 0.91 | -0.09 | 0 |
| MD.tr | 0.43 | 0.73 | 0.47 | -0.25 | 0.05 | 0 |
| BD.tr | 0.44 | 0.17 | -0.74 | -0.21 | -0.43 | 0.02 |
| TL.tr | 0.54 | -0.64 | 0.41 | -0.23 | -0.29 | 0.01 |
| BL.tr | 0.42 | -0.17 | -0.26 | -0.07 | 0.85 | 0.04 |
| Mass.tr | 0.03 | -0.01 | -0.02 | -0.01 | 0.02 | -1 |
